# Optimizing Diets for the Prevention of Cardiovascular Diseases and the Promotion of Cardiometabolic Health

**DOI:** 10.1093/nutrit/nuag054

**Published:** 2026-05-26

**Authors:** Cheryl A M Anderson, Nicole Karongo

**Affiliations:** Herbert Wertheim School of Public Health and Human Longevity Science, University of California San Diego, La Jolla, CA 92093-0628, United States; Herbert Wertheim School of Public Health and Human Longevity Science, University of California San Diego, La Jolla, CA 92093-0628, United States

**Keywords:** spices, culturally tailored, public health nutrition, cardiometabolic health, foodways

## Abstract

**Objectives:**

This article explores the role of herbs and spices as culturally grounded tools to support the adoption of high-quality diets and promote cardiovascular health.

**Background:**

Cardiovascular disease remains a leading cause of morbidity and mortality worldwide, with suboptimal dietary patterns contributing substantially to risk. Traditional dietary interventions often fail to account for the complex socioeconomic, cultural, environmental, and behavioral determinants that shape food choices.

**Methods:**

Descriptive data from randomized clinical trials and proof-of-concept studies that incorporate spices and herbs into healtful dietary patterns to reduce sodium intake and improve diet quality.

**Results:**

Evidence from the SPICE trial and ongoing HAWAASH studies demonstrates that behavioral interventions incorporating herbs and spices can effectively reduce sodium intake and encourage nutrient-dense food consumption.

**Conclusions:**

These findings provide practical insights for healthcare providers seeking to personalize dietary counseling and nutrition prescriptions, particularly for individuals at risk of or living with cardiometabolic diseases. Incorporating the cultural significance of herbs and spices into public health strategies may offer a promising avenue to enhance dietary adherence, respect traditional foodways, and advance equity in cardiovascular health promotion.

## HERBS AND SPICES AS A STRATEGY TO OPTIMIZE DIETS THAT PROMOTE CARDIOVASCULAR HEALTH

In the pursuit of effective strategies to combat cardiovascular disease (CVD), dietary interventions play a pivotal role. The SPICE trial, sponsored by the McCormick Science Institute (MSI) and published in the *American Journal of Clinical Nutrition* in 2015,^1^ explored the impact of an intensive behavioral intervention emphasizing spices and herbs on sodium intake adherence. The HAWAASH (Healthy Eating with Spices and Herbs to Manage Hypertension) studies,^2–4^ also sponsored by MSI, have an overarching aim to refine and optimize the diet for the prevention of chronic disease and the promotion of health equity across all populations.

Despite decades of public health efforts, there is persistent low adherence to the US Dietary Guidelines,^5^ and suboptimal diets remain a leading cause of morbidity and mortality globally.^6^^,^^7^ Traditional interventions, while valuable, often fail to account for the complex interplay of socioeconomic, cultural, environmental, and behavioral factors that influence food choices.^8^

## HERBS AND SPICES IN SHAPING HEALTHY DIETARY PATTERNS IN DIVERSE CULTURAL CONTEXTS

The role that herbs and spices play in shaping healthy dietary patterns across diverse cultural contexts is understudied. Our research team’s MSI-sponsored HAWAASH studies hypothesized that herbs and spices would be important and appreciated tools that could be culturally embedded in interventions to promote high-quality diets in East African immigrant populations for whom chronic disease risk reduction is warranted.

The HAWAASH studies’ contributions to improving the palatability of nutritious foods without reliance on excess sodium, added sugars, or unhealthy fats are particularly salient in interventions and campaigns aimed at cardiovascular health promotion. We partnered with individuals of East African heritage living in San Diego for whom culinary traditions rely on complex spice blends—such as berbere, mitmita, xawaash, chai and pilau masala, and ras-el-hanout—to enhance flavor. Our findings will guide the use of spices and herbs to support nutrient-dense, healthful meals that align with many evidence-based dietary guidelines.^9^

## INTERVENTIONS THAT EMPHASIZE SPICES AND HERBS THAT ARE INCLUSIVE AND EVIDENCE-BASED

Even when people know what to eat, changing long-term habits is difficult.^10^ Diet interventions can prevent or delay disease, improve quality of life, and reduce healthcare costs.^10^ Behavioral interventions can change individuals’ food intake, and when designed with attention to cultural relevance and accessibility, they may also reduce differences in health across groups.

### SPICE trial 

The SPICE trial was conducted in Baltimore, Maryland, and involved a 2-phase design.^1^ In phase 1, 55 participants adhered to a low-sodium diet for 4 weeks, receiving all meals and snacks provided by the study team. Phase 2, lasting 20 weeks, randomized 40 participants from phase 1 into 2 groups: a behavioral intervention group and a self-directed control group. The primary outcome measured was the change in 24-hour urinary sodium excretion, which served as a biomarker for sodium intake. Secondary outcomes included blood pressure, weight, and dietary adherence. In the SPICE trial, participants had a mean age of 61 years, with 65% women and 88% African American participants. Notably, 63% had hypertension and 18% had diabetes, reflecting a population at increased risk of CVD. This demographic is particularly significant, as African Americans experience higher rates of hypertension and related complications.^10^

**Figure 1.**
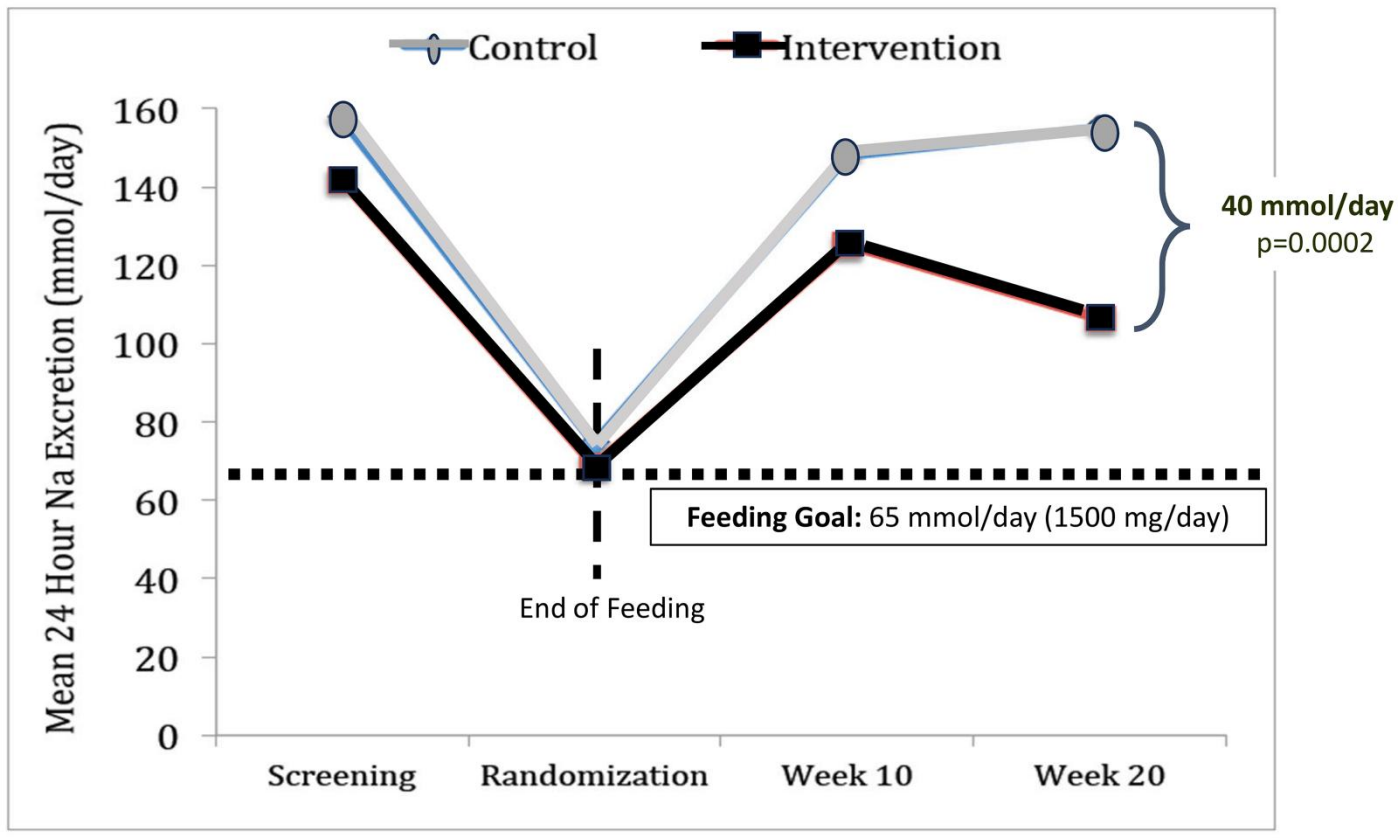
SPICE Intervention Lowers Sodium Intake Over Time

The SPICE behavioral intervention focused on reducing sodium intake by encouraging the use of spices and herbs as flavoring agents. Participants in the intervention group received education on the health risks associated with excessive sodium consumption and training on incorporating various spices and herbs into their meals. This approach aimed to enhance flavor without relying on salt, thereby improving dietary adherence to recommended sodium levels. At the conclusion of phase 2, the intervention group exhibited a significant reduction in 24-hour urinary sodium excretion compared with the control group. The mean difference was −956.8 mg/day (95% CI: −1538.7, −374.9 mg/day), indicating a substantial decrease in sodium intake (**Figure 1**). These findings persisted even after adjusting for potential confounders, underscoring the effectiveness of the intervention.

A unique feature of the SPICE trial was that the behavioral intervention followed a phase of controlled feeding. Consistent with findings from other controlled-feeding studies, such as the Dietary Approaches to Stop Hypertension (DASH)^11^ and DASH-sodium,^12^ mean 24-hour urinary sodium excretion decreased in phase 1 to levels recommended for those enrolled. Consistent with findings from other behavioral interventions^13–15^ for sodium reduction, in phase 2 the mean 24-hour urinary sodium excretion decreased significantly, more so for those in the active intervention group than for those in the control group. However, the unique dataset of the trial on maintenance of sodium intake at the level of 1500 mg/day provided novel information for public health efforts to address the complexities of food environments, in addition to individual efforts for behavior change.

### Ongoing HAWAASH Studies

**Figure 2.**
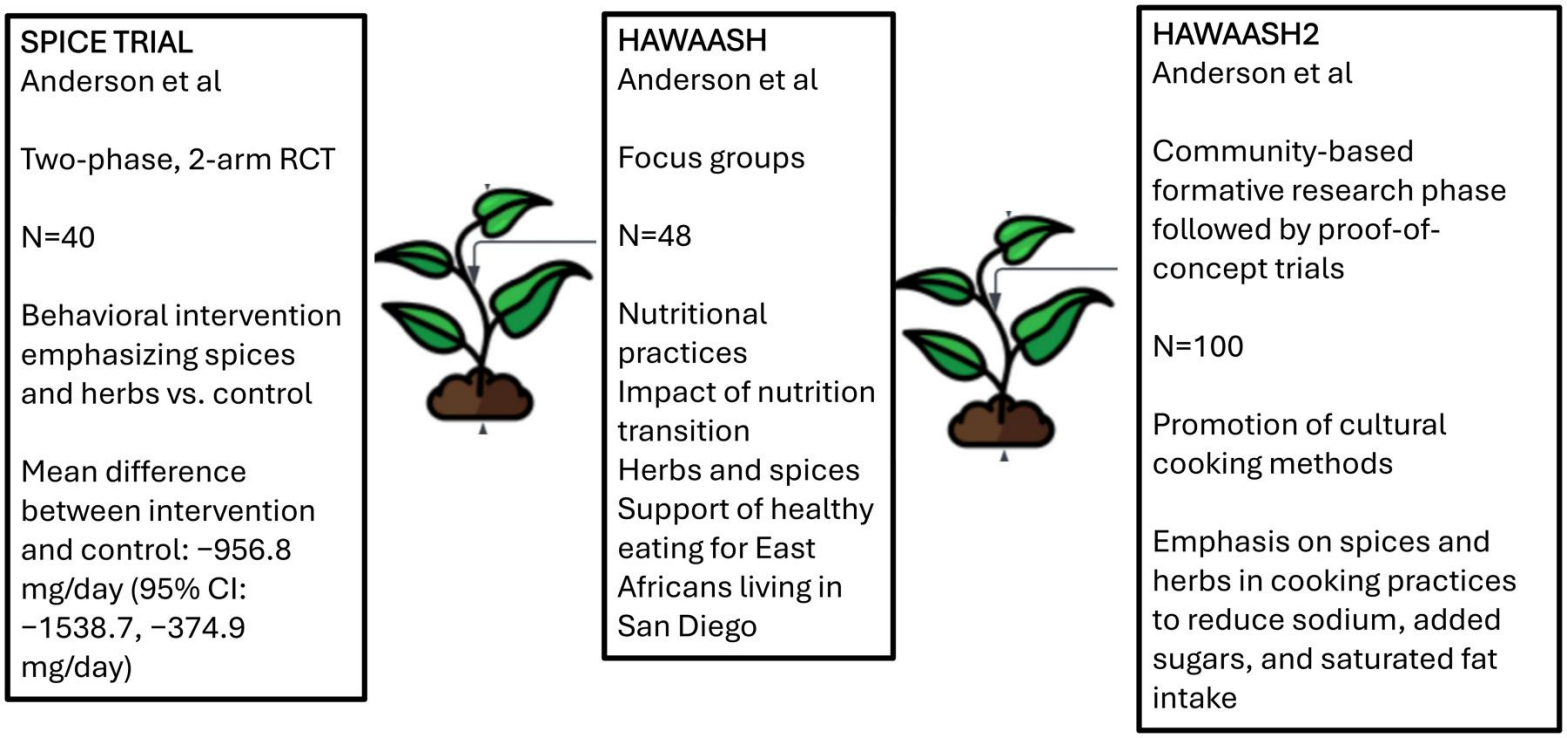
Summary of Design and Methods for the SPICE Trial and HAWAASH Studies Abbreviations: HAWAASH, Healthy Eating with Spices and Herbs to Manage Hypertension; RCT, randomized controlled trial.

With MSI’s sponsorship, we are conducting proof-of-concept trials^2–4^ to assess the feasibility and plausibility of a culturally tailored, nutrition-focused intervention to reduce sodium, added sugars, and saturated fat intake among East African immigrants in San Diego, with an emphasis on using herbs and spices as an accessible and acceptable strategy for behavior change (**Figure 2**). We are using a participatory, community-engaged approach that actively involves local residents, organizational stakeholders, and academic researchers throughout the entire intervention development process. Results from our formative interviews, co-design workshops, and proof-of-concept trials revealed that standard tools for assessing dietary intake and biometric outcomes can be appropriately adapted for an East African population when paired with culturally sensitive language, community-based settings, and involvement of trusted local partners. Community members expressed varying degrees of comfort with research procedures, such as the content of cooking classes and likelihood of incorporation into daily life. We observed a strong preference for in-person activities held at familiar locations such as the community-based offices of the United Women of East Africa (UWEAST) and facilitated by peers from their own communities. Our findings to date support the importance of culturally responsive and community-rooted strategies in nutrition research and highlight the utility of iterative design frameworks that incorporate stakeholder feedback and plan for long-term sustainability.

## PUBLIC HEALTH IMPORTANCE OF HEALTHY DIETARY PATTERNS THAT INCLUDE SPICES AND HERBS

**Figure 3.**
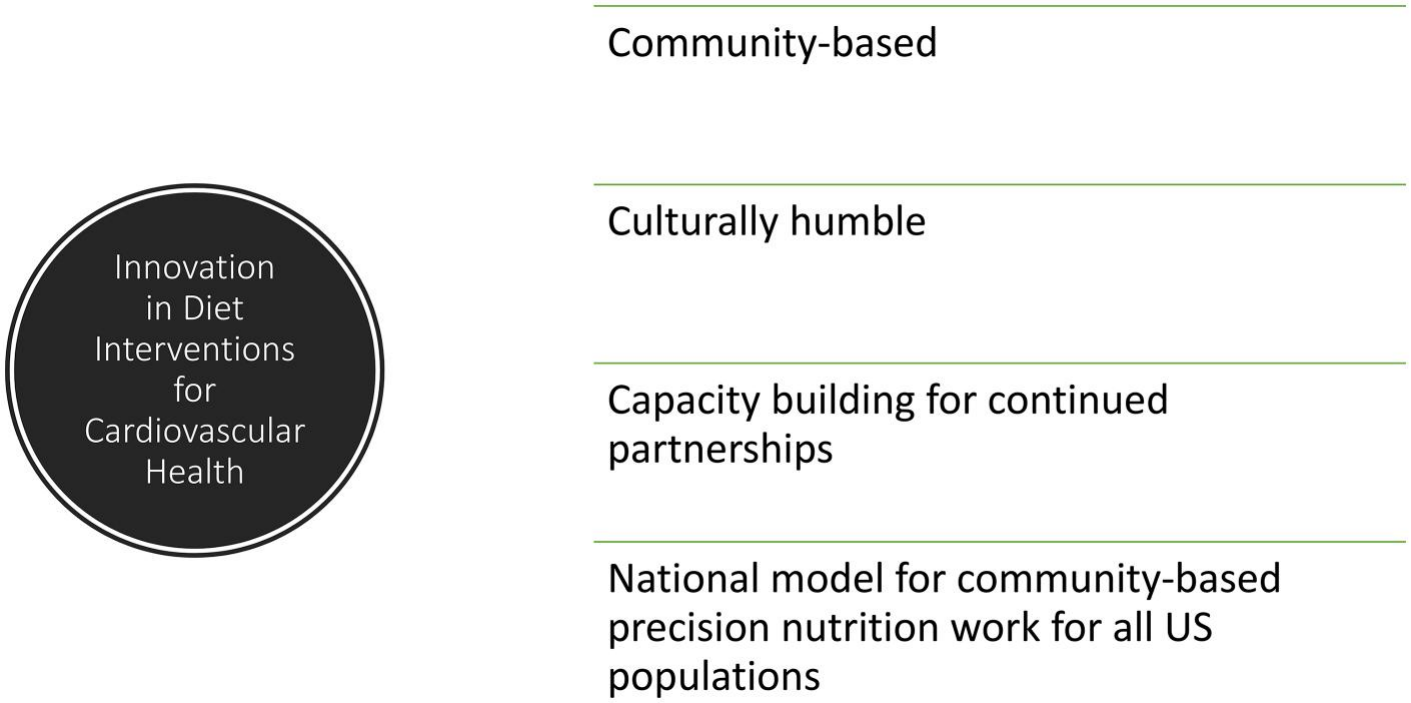
Innovation in Diet Interventions Emphasizing Spices and Herbs for Improving Cardiovascular Health

The SPICE trial’s and the ongoing HAWAASH studies’ findings are significant in the context of public health nutrition. Herbs and spices are deeply tied to cultural identity and food heritage. Recognizing their value allows for the development of innovative dietary interventions that are both cardiovascular health-promoting and culturally respectful, as public health strategies that incorporate traditional knowledge—rather than supplant it—are more likely to be adopted and sustained (**Figure 3**). Educational materials and cooking demonstrations that highlight the benefits and uses of spices and herbs can assist individuals in transforming nutritious foods into tasty meal options.

## CONCLUSION

The success of the SPICE trial and HAWAASH studies demonstrates clear, evidence-based strategies that healthcare providers can integrate into dietary counseling and nutrition prescriptions, particularly for individuals with cardiometabolic diseases. Our studies expand the research investments of MSI from cellular research into societal research. Behind every breakthrough in science are the participants and communities who engage with our work. With respect for this sentiment, our findings offer actionable, real-world data that community partners can scale and implement—laying the groundwork for success of food programs that are needed to support those who are medically vulnerable and those who seek to maintain health and prevent disease.

## References

[1] Anderson CAM , Cobb LK, Miller ER, et al Effects of a behavioral intervention that emphasizes spices and herbs on adherence to recommended sodium intake: results of the SPICE randomized clinical trial. Am J Clin Nutr. 2015;102:671-679. 10.3945/ajcn.114.100750 26269371 PMC4548171

[2] Karongo NN , Atun AE, Abdi S, et al Characteristics of dietary behaviors and effects of dietary acculturation in East African immigrants living in the San Diego area: preliminary insights from the HAWAASH2 study. Poster presented at: 2024 Food & Nutrition Conference & Expo (FNCE^®^); October 5-8, 2024; Minneapolis, Minnesota.

[3] Karongo NN , Atun AE, Abdi S, et al Characteristics of dietary behaviors and cardiometabolic health in East African Immigrants living in the San Diego area: preliminary insights from the HAWAASH2 study. Poster presented at: American Society of Nutrition (Nutrition 2024); June 29-July 2, 2024; Chicago, Illinois.

[4] Karongo NN , Atun AE, Abdi S, et al Characteristics of dietary behaviors and effects of dietary acculturation in East African immigrants living in the San Diego area: preliminary insights from the HAWAASH2 study. Poster presented at: American Public Health Association (APHA); October 27-30, 2024; Minneapolis, Minnesota.

[5] Dietary Guidelines Advisory Committee. Scientific Report of the 2025 Dietary Guidelines Advisory Committee: Advisory Report to the Secretary of Health and Human Services and the Secretary of Agriculture. US Department of Agriculture, Agricultural Research Service, Washington, DC. 2025. Accessed May 26, 2025.https://www.dietaryguidelines.gov/2025-advisory-committee-report

[6] GBD 2017 Diet Collaborators. Health effects of dietary risks in 195 countries, 1990–2017: a systematic analysis for the Global Burden of Disease Study 2017. Lancet. 2019;393:1958-1972. 10.1016/S0140-6736(19)30041-8 30954305 PMC6899507

[7] Afshin A , Penalvo J, Del Gobbo L, et al Dietary policies to improve population health: a global perspective. PLoS Med. 2015;12:e1001842. 10.1371/journal.pmed.1001842 26103555 PMC4477873

[8] Development Initiatives. *2018 Global Nutrition Report: Shining a Light to Spur Action on Nutrition*. Development Initiatives; 2018.

[9] Anderson CAM , Murray KE, Abdi S, et al Community-based participatory approach to identify factors affecting diet following migration from Africa: the HAWAASH study. Health Educ J. 2019;78:238-248. 10.1177/0017896918814059

[10] Martin SS , Aday AW, Allen NB, et al; American Heart Association Council on Epidemiology and Prevention Statistics Committee and Stroke Statistics Committee. Heart disease and stroke statistics: a report of US and global data from the American Heart Association. Circulation. 2025;151:e41-e660. 10.1161/CIR.0000000000001303 39866113 PMC12256702

[11] Appel LJ , Moore TJ, Obarzanek E, et al A clinical trial of the effects of dietary patterns on blood pressure. DASH Collaborative Research Group. N Engl J Med. 1997;336:1117-1124. 10.1056/NEJM199704173361601 9099655

[12] Sacks FM , Svetkey LP, Vollmer WM, et al; DASH-Sodium Collaborative Research Group. Effects on blood pressure of reduced dietary sodium and the Dietary Approaches to Stop Hypertension (DASH) diet. N Engl J Med. 2001;344:3-10. 10.1056/NEJM200101043440101 11136953

[13] Appel LJ , Champagne CM, Harsha DW, et al; Writing Group of the PREMIER Collaborative Research Group. Effects of comprehensive lifestyle modification on blood pressure control: main results of the PREMIER clinical trial. JAMA. 2003;289:2083-2093. 10.1001/jama.289.16.2083 12709466

[14] Trials of Hypertension Prevention Collaborative Research Group. The effects of nonpharmacologic interventions on blood pressure of persons with high normal levels: results of the Trials of Hypertension Prevention Phase 1. JAMA. 1992;267:1213-1220. 1586398 10.1001/jama.1992.03480090061028

[15] Trials of Hypertension Prevention Collaborative Research Group. Effects of weight loss and sodium reduction intervention on blood pressure and hypertension incidence in overweight people with high-normal blood pressure: the Trials of Hypertension Prevention, Phase II. Arch Intern Med. 1997;157:657-667. 9080920

